# Do Healthy City Performance Awards Lead to Health in All Policies? A Case of Taiwan

**DOI:** 10.3390/ijerph16061061

**Published:** 2019-03-24

**Authors:** Nuan-Ching Huang, Hsien-Wen Kuo, Te-Jen Hung, Susan C. Hu

**Affiliations:** 1 Healthy Cities Research Center, Research and Services Headquarters, National Cheng Kung University, No.1, University Road, Tainan City 701, Taiwan; sunnynching@gmail.com; 2 Institute of Environmental and Occupational Health Sciences, National Yang-Ming University, No.155, Sec.2, Linong Street, Taipei 112, Taiwan; hwkuo1106@gmail.com; 3 BeiTou Culture Foundation, No.45-1, Sec. 1, Zhongyang S. Rd., Beitou Dist., Taipei City 112, Taiwan; hungtj66@ms31.hinet.net; 4 Department of Public Health, College of Medicine, National Cheng Kung University, No.1, University Road, Tainan City 701, Taiwan

**Keywords:** healthy cities, innovation performance awards, intersectoral collaboration, health in all policies

## Abstract

The Healthy Cities (HC) Project, which was introduced by the World Health Organization (WHO) in 1986, has been recognized as the best setting approach for health promotion. However, very few studies have addressed how to use HC approaches to establish public policies in non-health departments in cities. This paper describes the strategies for the HC Performance Awards used in Taiwan to draw attention from different departments and to sustain intersectoral collaboration for the purpose of establishing Health in All Policies (HiAP). The methods include: (1) setting up the Taiwan Healthy City Alliance; (2) establishing HC Innovation Performance Awards; (3) reviewing the award applications according to seven criteria; and (4) analyzing the topic content of the award applications. We collected 961 HC award applications during 2013–2016 to analyze their content. The results showed that the number of applications increased nearly every year while significantly more non-health departments applied for the awards compared to health departments (73.3% vs. 26.7%). The award rates of non-health departments have also increased twice from 13.9% in 2013 to 25.8% in 2016. By examining the topics of the award winners, we concluded that “HC Innovation Performance Awards” indeed provide a role and opportunity for political involvement, intersectoral collaboration, co-opetition and capacity building that is necessary for establishing health in all policies.

## 1. Introduction

The healthy city (HC) movement is the best-known settings-based approach to health promotion, which is aimed towards making health a high priority on the agendas of decision makers and promoting comprehensive local strategies that lead to health promotion and sustainable development [1]. Community participation and empowerment, intersectoral partnership and collaboration and equity among diverse groups are the basic features for promoting healthy cities. These features engage local governments in health development through a process of political commitment, institutional change, capacity-building, partnership-based planning and innovative projects [2]. HC projects have six characteristics in common: (1) commitment to health; (2) political decision-making; (3) intersectoral action; (4) community participation; (5) innovation; and (6) healthy public policy [2]. Specifically, the final characteristic, which is namely healthy public policies, can be achieved through the other five above-mentioned approaches [2]. In addition, a national network also plays an important role in the dissemination of HC strategies and the building of communication between cities at the national level.

### 1.1. Health in All Policies (HiAP)

However, many policies from non-health departments, such as construction, transportation, public safety, urban planning, housing, education, employment and so on, occasionally result in health problems and burdens. Therefore, integrating health considerations in public policies across different departments will affect various settings in which people live and work [3]. Thus, the Finnish Presidency of the European Union (EU) proposed the Health in All Policies (HiAP) concept in 2006, which is defined as “an approach to public policies across sectors that systematically takes into account the health implications of decisions, seeks synergies and avoids harmful health impacts, in order to improve population health and health equity” [4].

Nevertheless, HiAP is an abstract concept with rhetorical ideas. It is a challenge to convert this abstract concept into practice and evaluation [5,6]. Policy formulation is both an approach and a goal or a strategy and subsequently, its development and establishment is not a single linear process. It is an iterative, dynamic process in which multiple situations will arise and where there will be a need to negotiate with many stakeholders [7]. Therefore, determining how to promote such programs and formulating strategies to involve different sectors in terms of collaboration, implementation and sustainability of the mechanism for HiAP is still an important concern [6,8,9,10].

### 1.2. Strategies Used in HC Projects and HiAP

Four basic strategies for HiAP practice have been identified: (1) a health strategy; (2) a win-win strategy; (3) a cooperation strategy and (4) a damage limitation strategy [5]. In South Australia, the key mechanism of HiAP is a process called a Health Lens Analysis (HLA), which includes the five stages of engagement, evidence gathering, generating, navigating and evaluating. The HLA has been designed to shift the policy frame and inform the policy at the conceptual stage rather than towards the end of decision-making processes [11]. Furthermore, Freiler et al. [12] proposed that HiAP includes two parts, which are namely agenda setting and capacity building. The strategies used in these two parts include (1) agenda setting; (2) raising awareness; (3) a win-win approach; (4) capacity building; (5) institutional capacity; (6) expert capacity; and (7) prior experience [12]. Thus, Gase et al. proposed seven interrelated strategies for incorporating health considerations into decisions and systems: (1) developing and structuring cross-sector relationships; (2) incorporating health into decision-making processes; (3) enhancing workforce capacity; (4) coordinating funding and investments; (5) integrating research, evaluation and data systems; (6) synchronizing communications and messaging; and (7) implementing accountability structures [13].

In summary, the goal to enhance health awareness, capacity building, intersectoral communication, collaboration for stakeholders and political tasks (win–win) is common to all these strategies. However, it is very difficult to measure the status and results of HiAP development due to the complexity, limitations of practical experience and political backgrounds involved [7,14]. Up to the present, very few studies have focused on how to put political involvement in HC projects and HiAP into practice.

### 1.3. Objective

The Shanghai Declaration on Health Promotion in 2016 calls again for good governance of health issues and support for healthy cities and communities [15]. Therefore, the objective of this paper is to describe the strategies used for the HC Performance Awards in Taiwan, which are intended to maintain HC project achievements, sustain the intersectoral collaboration mechanism and achieve the goals of HiAP.

## 2. Methods

The methods used in this study include (1) setting up the Taiwan Alliance for Healthy Cities; (2) establishing the Healthy City Innovation Performance Awards; (3) reviewing the Innovation Performance Award applications across seven criteria; and (4) collecting and analyzing the topic content of the award applications.

### 2.1. Setting up the Taiwan Alliance for Healthy Cities (TAHC)

The Healthy Cities Projects in Taiwan started in 2003. Subsequently, more cities and counties have devoted themselves to the promotion of healthy cities. On February 2006, the fellows in the Healthy City Research Center, National Cheng Kung University suggested that the Minister of the Department of Health and the Mayor of Tainan City should serve as conveners; the Mayors of Taipei and Kaohsiung City should serve as co-conveners and that all county and city mayors in Taiwan should be invited to participate in the Taiwan Healthy Cities Network Summit. At the summit, around 70 participants, including mayors, deputy mayors and representatives from 23 counties and cities, all drafted and signed the Protocol for Healthy Cities, at which time they agreed to hold annual meetings to discuss a yearly workshop to promote the healthy cities projects.

Therefore, the Taiwan Alliance for Healthy Cities (TAHC), a non-government organization, was officially established on January 2008, which aimed to facilitate communications and collaboration among local governments, related departments, scholars and experts, private institutions and community organizations. This organization was created for the purpose of establishing mutually beneficial partnerships that are intended to help achieve the goals of healthy cities. The members of TAHC were divided into two levels: (1) city and county level and (2) township level. Until 2018, the members of TAHC were comprised of 20 (95.2%) cities and counties in Taiwan. The TAHC has three commissions and their responsibilities are divided as follows: (1) a research and development group for collecting, monitoring and evaluating of city indicators; (2) an events and training group for planning education and training programs related to healthy cities; and (3) an award evaluation group for setting and selecting awards for HC programs. The detailed descriptions of the mechanism and the process of creating a healthy city in Taiwan have been published in another article [16].

### 2.2. Establishing the Healthy City Innovation Performance Awards

In order to encourage local governments to devote themselves to establishing Healthy Public Policy and promoting the concept of Health in All Policies into practice, the TAHC established the Healthy City Innovation Performance Awards as a strategy for intersectoral collaboration in order to actively integrate resources and programs that are intended to demonstrate involvement and achievements. In the case of non-health departments, this particularly aims to highlight the critical role of non-health departments in establishing “Health in All Policies.” All award winners can demonstrate their achievements in the award ceremony annually through oral presentations and field visits.

The Taiwan Healthy City Innovation Performance Awards, which were designed based on the concept of an idea healthy city [2], were first announced in 2009 and include nine items: (1) health promotion policy; (2) healthy environments; (3) healthy living; (4) sustainability; (5) industrial development; (6) mental health; (7) safety and security; (8) equity; and (9) comprehensiveness.

### 2.3. Reviewing the Innovation Performance Awards Applications

The review of the Innovative Performance Award applications includes two stages. First, all applications in each item are reviewed and graded by three experts. Second, all grades, comments and rankings of the reviewers in the first stage are examined again by five additional experts (to avoid conflicts of interest) to confirm the final winners. The award criteria for each item include:

- Background of the issue (10%): Please describe the background information and the importance of the topic issue in your city/county.
- How to promote this issue and its innovation (20%): Please describe the strategies and steps for promoting the issue and point out the innovation and implication of the approaches as well as the effectiveness of the results.
- Mechanisms for intersectional cooperation (20%): Please describe the mechanism and structure of working with other departments and how to evaluate your collaboration effects.
- Civic participation and its effects (10–15%): Please describe the process and results for community participation and how to connect with healthy city projects.
- Related outcomes and effectiveness (20%): Please describe your project outcomes, including qualitative and quantitative results, such as the change of related indicators.
- The mechanisms of sustainability and monitoring (10%): Please describe the mechanisms for sustainable development and how to establish a regular monitoring and review process.
- Other innovative effects (10%): Please add any other distinguishing features or approaches to the promotion of this issue and describe the popularity and applicability of the above innovations to other districts or cities.

At most, five outstanding applications for each item are awarded in the award ceremony. The President or Vice President of Taiwan or the Secretary-General or Deputy Secretary-General of the presidential office are invited to the award ceremony as the awarder to raise the level of the HC awards. As a result, the winners of the department heads feel proud and honored to receive this recognition from a top governmental leader. TAHC encourages winners to rewrite their application documents in English according to the reviewers’ comments to apply for other international Alliance for Healthy Cities (AFHC) awards and to exchange their experiences with other international cities in the Asia Pacific region.

### 2.4. Collecting and Analyzing the Content of the Award Applications

In order to evaluate HiAP achievement through the Innovative Performance Awards, 961 related documents were collected for the purpose of this study, including committee reports, application forms and award topics in the Innovative Performance Awards during 2013–2016. We first analyzed the number of applications and growth rate during the eight years under observation before examining the award rates between health and non-health departments and their focus in the applications. As the award items and evaluation criteria have been modified during 2009–2012 and remained stable since 2013, we only analyzed the results and topics of applications during 2013–2016 in this study. Finally, we selected some winning topics as examples to explain the importance and implications of intersectoral collaboration for HiAP.

## 3. Results

### 3.1. Number of Applications and Award Rates

**Figure 1:**
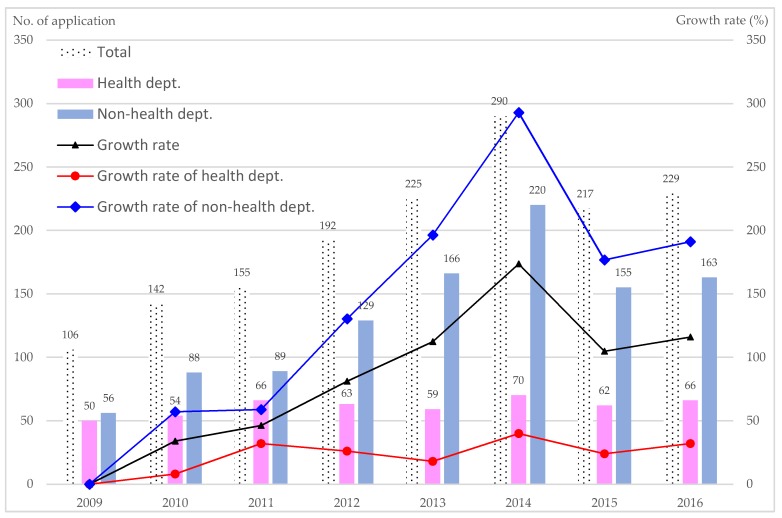
Number of applications per year and growth rates. Growth rate (%) = [(no. of application in 2010~2016) − no. of application in 2009] ÷ no. of application in 2009 × 100%.

Figure 1 presents the number and growth rate for the award applications during 2009–2016. The number of applications increased annually. The application growth rate reached its highest (173.6%) in 2014 (black line). Because the number of applications has become a burden for the reviewers who have to review all of the applications in a limited time period, the TAHC added a new rule after 2014 for award applications: the applications for each item were limited to a total of three for each member city. Therefore, the total number of applications and the growth rate dropped in 2015 but still increased slightly in 2016. Noticeably, the number of award applications and growth rate of non-health departments (blue color) are higher than that in health departments (red color) during 2012–2016.

**Figure 2:**
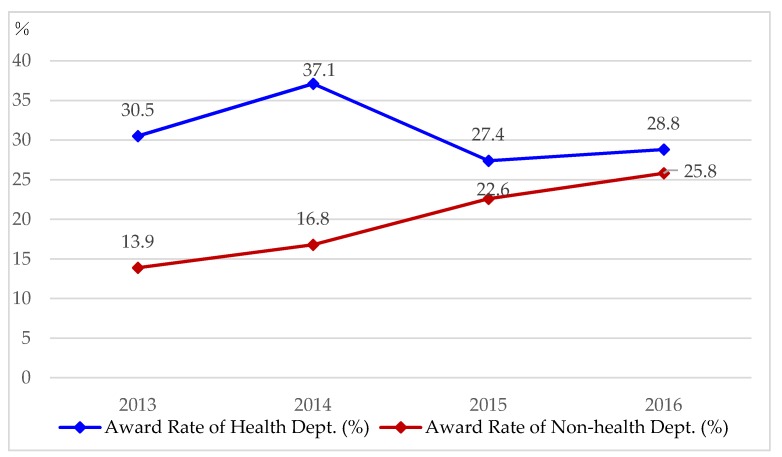
A comparison of award rates for health and non-health departments during 2013–2016. Award rate of health dept. = no. of winners in health department/no. of applications in health department × 100%. Award rate of non-health dept. = no. of winners in non-health departments/no. of applications in non-health departments × 100%.

Figure 2 shows a comparison of the award rates for the health and non-health departments (the details of this are provided in the Appendix A Table A1). The award rate for the health department was higher than that for the non-health departments. However, the award rate for non-health departments increased nearly two-fold from 13.9% in 2013 to 25.8% in 2016.

### 3.2. The Popular Items and Award Rates Between Health and Non-Health Departments

**Figure 3:**
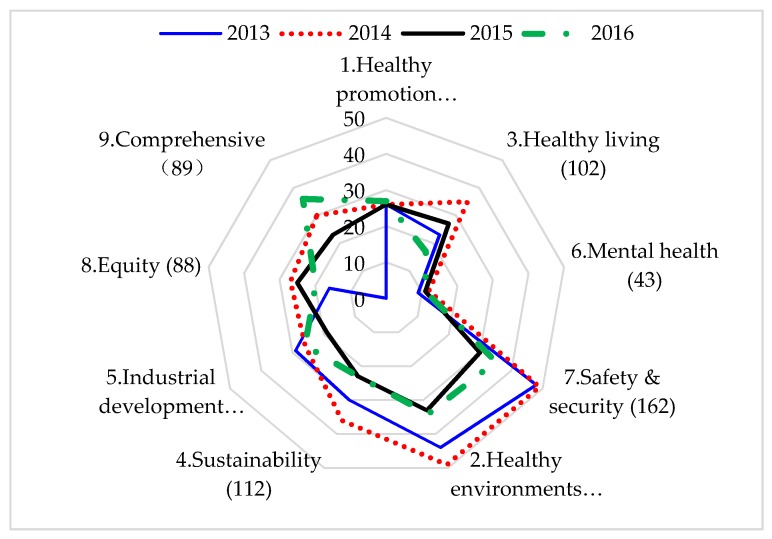
Award items and total no. of applications per item during 2013–2016.

Among the 961 award applications, “safety and security” was the most popular item during 2013–2016. As shown in Figure 3, the five most popular items and their total application numbers in order were safety and security (162 applications); healthy environments (160 applications); sustainability (112 applications); health promotion policy (105 applications); and healthy living (102 applications). However, “mental health” was the item with the fewest applications. It is noted that for some items, such as “safety and security” and “healthy environments”, the number of applications decreased during 2013–2016. This might create the challenges for sustaining innovations for the short-term results of some topics. In contrast, although there have been more challenges associated with promoting the long-term issues, such as “health promotion policy” and “mental health”, the number of applications remained stable.

Table 1 provides a comparison of the number of applications for the health and non-health departments. Compared with the non-health departments, health departments focused more on “health promotion policy” and “mental health” items (*p* < 0.0001). On the other hand, the top three items on the applications from non-health departments were safety and security, healthy environments and sustainability.

Table 2 illustrates the results for the total number of applications and winners during 2013–2016 by item. The top three award rates for health departments were sustainability, mental health and healthy living. As for the non-health departments, the top three items were mental health, comprehensive and industrial development. However, the number of applications from health departments for sustainability and those from non-health departments for mental health were very few, with only 2 and 14 applications, respectively.

### 3.3. Examples of Winning Topics

We selected four items and 40 winning topics in Table 3 to observe what types of issues existed in the health and non-health departments that the local government focused on and practiced well. First, in the health promotion policy item, the winning topics from health departments included cancer screening, eye care, smoking, food safety, safe sex and physical fitness. However, the winning topics from non-health departments included early screening, school lunch, ageing in place, food safety and child health. It should be noted that the topics for both departments were similar but the winners in the non-health departments came from many different departments, such as social affairs, education, legal affairs and environmental protection. They established health promotion policies and integrated them into different settings or special populations, such as children, older adults and general consumers. In particular, some specific topics upgraded the level of programs into regulations, such as setting protective screening for consumer food safety in Changhua and establishing self-governance ordinance for a low-carbon city (low carbon, healthy Tainan project).

Second, for the mental health item, most winning topics focused on depression, consultation and suicide prevention. It should be noted that the non-health departments were diverse, including bureaus of civic affairs, social affairs and personnel who highlighted the importance of mental health in special populations, such as the military, public staff and abused children. All these are the so-called “hard to reach” groups and with the help of non-health departments, their involvement could really lower work stress in health departments.

As to healthy environment items, most topics from health departments considered the establishment of smoking-free environments. However, it is impressive that some health service organizations encompassed certain items, such as the connection of sidewalk and roads for cycling, walking, healthy eating and sightseeing among health issues. The topics from non-health departments were also diverse and included river remediation, green infrastructure, landscape improvement, urban regeneration, rural revitalization and other physical infrastructures. These topics are all related to the determinants of health in a livable city.

Finally, the topics related to the equity item for the health departments included tuberculosis care, care access, health examinations or vaccine use for at-risk populations. However, a majority of topics from the non-health departments addressed the rights of and assistance for disabled or vulnerable populations and new immigrants, including access to related services, employment, healthcare, physical activities and reading practice. All these topics involved equal opportunity and health inequality among different subgroups.

## 4. Discussion

In this study, the results showed that the national network, the TAHC, serves as a good platform to integrate participation in both the vertical and horizontal dimensions of government agencies, municipalities, academia, community organizations and citizens. It also helps to build a sustainable capacity and climate in addition to providing motivation for cities or counties to promote HC projects and establish related public policies. In addition, the TAHC was set up as a non-governmental organization (NGO) so that it could sustain its ideas and missions and avoid any uncertain effects from political and administrative transitions.

The advantages of the awards in promoting the HC program are included in another published article [16]. This study focused on analyzing the participation rates and topic contents of HC awards between health and non-health departments in order to help improve the determinants of health within a city. The number of applications and award rates of non-health departments increased nearly two-fold during 2013–2016, which shows that the establishment of the HC Innovative Performance Awards not only encouraged non-health departments to become involved in the promotion of healthy cities through intersectional collaboration but also highlights the achievements resulting from their participation in HC projects. By examining the topics of award winners, we found that the TAHC and the Healthy City Innovation Performance Awards have complementary roles and provide opportunities to exhibit: (1) political achievements; (2) intersectoral collaboration; (3) co-opetition in terms of competition and collaboration; and (4) capacity building. All four of these components are the essential approaches for establishing Health in All Policies.

### 4.1. Political Achievement

The political stream is an essential factor that must be considered when promoting HC and HiAP [17,18,19]. Politics is about the distribution of power and resource distribution as well as the management of conflicting interests in order to bring about and maintain social order and cohesiveness. Political transitions, such as portfolio reshuffles, political and administrative changes and bureaucratic staff turnover, tend to affect the promotion of related projects and policies [20]. The TAHC and Healthy City Innovation performance Awards are a formal platform that is used to present both the commitment and achievements of politically-driven organizations.

### 4.2. Intersectoral Collaboration

Intersectoral collaboration is another essential element that is used to promote HC projects and HiAP [2,4,13,17,21]. Health is determined and impacted by many environmental factors related to living and working, including social, economic, physical and environmental ecosystems [21,22]. Many major health negotiations and the development of related policies have been led by experienced diplomats rather than health experts [23]. Therefore, enabling, mediating and advocating non-health departments to promote HC projects can be instrumental in advancing HiAP [23]. Scholars and HC core groups should help local governments to set up mechanisms for enabling intersectoral collaboration for HC promotion. However, simply depending on the connections within local governments makes it difficult to sustain motivation and provide a sense of freshness to HC projects. Thus, the TAHC and Innovative Performance Awards provide another, higher level of incentive.

For example, the Innovative Performance Awards are a good mechanism for considering both political issues and intersectoral collaboration. Items in the Innovative Performance Awards are full of variety and are based on the eleven idea qualities of HC [2]. The award applications are not at the beginning of a proposed stage as the projects must have been implemented for a while and demonstrated successful outcomes, effectiveness. Furthermore, there is rigorous assessment to determine their levels of sustainability, maintenance and evaluation. Therefore, when a city or a county wins awards for more than one item, partial credit should be given to the Mayor. These winning topics are announced and awarded by the President or Vice President of Taiwan or the Deputy Secretary-General of the presidential office. The HC awards ceremony is also a good place to present policy outcomes, publicize achievements and enhance political visibility. The strategies used in previous studies rarely have taken political visibility and public recognition into consideration [7,13,17,21].

### 4.3. Co-Opetition (Competition and Collaboration)

Many studies have indicated the importance of intersectoral collaboration in promoting HC activities and HiAP [5,21,24]. However, for a long time, different sectors and departments of local governments have always competed rather than collaborated with each other. Therefore, determining how to bridge the gap between competition and collaboration and setting up a win–win situation is the core of the HC Innovative Performance Awards.

The TAHC executive committee indeed provides an opportunity and a platform for co-opetition (competition and collaboration). First, half of the executive committee members of the TAHC are re-elected every two years. Hence, there is a competition in which the representatives of local governments can be selected as a member of the executive committee so they can participate in the affairs of the TAHC and contribute to making policies at the national level. Second, the HC Innovative Performance Awards create another opportunity and mechanism for different departments of local governments to compete and collaborate. The HC Awards require all applicants to establish intersectoral collaboration to promote healthy public policies in different dimensions, such as mental health of the army and government staff. When they want to win the award, every applicant has to compete with others, not only with those in other departments but also with those from other cities and counties. Therefore, the Innovative Performance Awards comprise a good strategy that combines co-opetition and public recognition to enhance the participation of non-health departments at the local government level. In addition, the media would report and announce the award results to compare the exertion of mayors and the advantages to the residents. Thus, it is also a co-opetition at the national level. Hence, it is a competition strategy and also a win–win strategy [8,21].

### 4.4. Capacity Building

Capacity building [17], expert capacity, workforce capacity [12] and prior experience [13] play the same role in enhancing capacity at different levels for HC promotion and HiAP. At the local government level, the steering committee of a healthy city will organize many training courses and discussion meetings for participants. At the national level, the TAHC also holds workshops on capacity building for HC promoters at different stages. For example, the TAHC holds both basic and advanced training workshops every year, where experts and scholars are invited to provide updated information and discuss future progress worldwide. The innovation award ceremony also provides an occasion for experience sharing and communication because the winners of awards will be invited to share their strategies, mechanisms and strategies. In addition, the TAHC arranges site visits at the training workshops and the award ceremony so the participants can learn, ask questions and directly see substantial changes in related policies. Some challenge items, such as mental health, need long-term discussion and preparation to implement a suitable program. However, it is difficult to achieve a substantial result in this type of topics in a short-term period. Usually, local governments are less willing to address and promote such topic items. Thus, we could use these award applications in training courses to build up the capacity for program organization and result presentation.

Briefly, the TAHC and HC Innovative Performance Awards have provided many different types of support and connections for intersectoral collaboration, capacity building, co-opetition, achievement exhibition and experience exchange for HC results and HiAP.

### 4.5. Contribution, Limitation and Future Suggestions

Although the concept of Health in All Policies (HiAP) was proposed in 2006, it is still a challenge to convert it into practice. The integration of health considerations in public policies across different departments is a very difficult task. Very few articles have addressed the experiences regarding HC projects and health in all policies in Asia. This is the first study to share the results of using HC awards as an approach to encourage non-health departments to participate in HC projects, which in turn could help to build up healthy public policy and reach the goals of HiAP.

However, some limitations need to be addressed in the study, including a lack of detailed information for newly-established intersectoral collaboration and community organization. Furthermore, determining how to completely present the effects of the four functions of HC projects (political achievements, intersectoral collaboration, co-opetition and capacity building) qualitatively and quantitatively is warranted in future studies.

Regarding the suggestions for Taiwan HC awards, there might be three directions for improvement. First, the items of HC awards could be re-designed into two levels, which are namely basic and advanced levels, in the future in order to accommodate different levels of development in various cities and communities. Second, the items of awards might be considered in more issues relating to international development, such as sustainable development goals (SDGs). Finally, the award could be added with some items related to HC ideas only, including proposals or protocols with the evaluation of the progress and results in the following years.

## 5. Conclusions

Taiwan has been devoted to HC projects for 15 years, which is not a long time in the grand scheme of things. Governments, community organizations and scholars in Taiwan still have exerted great effort to assist the TAHC in facing future challenges. The suggestions from Leonard Duhl and Trevor Hancock in 2004 were very helpful for promoting HC projects and related policies, such as national networks, urban planning and political tasks. Based on the experiences of Taiwan, the establishment of the TAHC and the HC Innovative Performance Awards are good approaches and mechanisms for promoting HC and related policies.

In conclusion, healthy city performance awards indeed provide a role in and opportunities for political involvement, intersectoral collaboration, co-opetition and capacity building for establishing health in all policies. 

## Figures and Tables

**Table 1:** No. of applications fom health or non-health departments during 2013–2016 by item.

Award Items	Total	Health Dept.	Non-health Dept.	*X*^2^ = 567.58	*p* < 0.0001
	*n* (%)	*n* (%)	*n* (%)		
1. Health promotion policy	105 (10.9)	66 (25.7)	39 (5.5)		
3. Healthy living	102 (10.6)	58 (22.6)	44 (6.3)		
6. Mental health	43 (4.5)	29 (11.3)	14 (2.0)		
7. Safety & Security	162 (16.9)	19 (10.9)	143 (20.3)		
2. Healthy environments	160 (16.6)	28 (17.5)	132 (18.8)		
4. Sustainability	112 (11.7)	2 (0.8)	110 (15.6)		
5. Industrial development	100 (10.4)	9 (3.5)	91 (12.9)		
8. Equity	88 (9.2)	31 (12.1)	57 (8.1)		
9. Comprehensiveness	89 (9.3)	15 (5.8)	74 (10.5)		
Total	961 (100)	257 (26.7)	704 (73.3)		

**Table 2:** The award rates of health and non-health departments from 2013–2016 by item.

Award Items	Health Department			Non-health Department			χ^2^	*p*
	No. of Applica.	No. of Awards	Award Rate (%)	No. of Applica.	No. of Awards	Award Rate (%)		
1. Health promotion policy	66	17	25.8	39	8	20.5	0.37	0.5421
3. Healthy living	58	21	36.2	44	3	6.8	12.01	**0.0005**
6. Mental health	29	13	44.8	14	5	35.7	0.32	0.5703
7. Safety and Security	19	5	26.3	143	25	17.5	0.87	0.3517
2. Healthy environments	28	5	17.9	132	25	18.9	0.02	0.8940
4. Sustainability	2	1	50.0	110	22	20.0	1.08	0.2980
5. Industrial development	9	2	22.2	91	20	22.0	0.00	1.000
8. Equity	31	11	35.5	57	12	21.1	2.17	0.1411
9. Comprehensiveness	15	5	33.3	74	17	23.0	0.72	0.3963

The bold number means that the result is statistically significant.

**Table 3:** Selected topics of winners for the innovation performance award.

Topics of Award Winners	Department
Health promotion policy	
(1) Initiatives for school lunch: Dietary education promotion at elementary and junior high schools	Education
(2) Food safety management in schools: Helping children eat natural and safe food	Education
(3) Setting protective screening for consumers: Food safety in Changhua	Legal Affairs
(4) Building a friendly city for pregnant women in Kaohsiung	Social Affairs
(5) Aging in place and LOHAS (Lifestyle of Health and Sustainability): the project for promoting health, happiness, safety and housing services for older adults	Social Affairs
(6) Love more in Kaohsiung: early treatment services delivery system for children with disability	Social Affairs
(7) Low carbon, healthy Tainan: Establishing self-governance ordinance for a low carbon city in Tainan	Environmental Protection
(8) Integrate screening for health; the results would be better!	Public Health
(9) Improving myopia together: building partnership for eye care for children	Public Health
(10) No smoking in youth	Public Health
(11) Improvement policy for the safety and sanitation of turkey rice in Chiayi City	Public Health
(12) Love in Taipei: The project for safe sex and healthy love	Public Health
(13) Healthy fitness: Enjoy being thinner program	Public Health
Mental health	
(1) Providing love to warm the broken hearts: Mental health services for children who witnessed family violence in Hsinchu City	Social Affairs
(2) Stopping the intergeneration transmission of child abuse: Early treatment for the parent–child relationship	Social Affairs
(3) Joining the army happily: safe, convenient and friendly military services	Civic Affairs
(4) Happiness starts from the heart: accessible, available, reliable and useful care services	Personnel
(5) Finding direction from your heart: The staff assistance project in Tainan City government	Personnel
Healthy Environments	
(1) Smoking outside at designated areas is a good approach to reduce the health risk of secondhand smoke	Public Health
(2) Live safely and leisurely in Jian	Public Health
(3) Go for a smoking-free and healthy Taipei: Effects of collaboration of public and private sectors	Public Health
(4) Building smoke-free sidewalk and eating delicious foods: Community building for different physical activities and new food cultures in Beitou	Health services center
(5) LOHAS in Hakka village and enjoying cycling	Civic Affairs
(6) Dating in the Jhongdou wetlands: Exploring ecological miracles in Kaohsiung	Public works
(7) Beautifying Caogong Ditch: pulsating water and a green river	Water Resource
(8) Making a good living in a station area: The high speed rail station special district projects in Houlong, Miaoli	Water Resource
(9) Riverside building for low-carbon and LOHAS New Taipei City Metropolitan Park	Water Resource
(10) Space building for artesian areas in Meinong Zhong-zhuang community	Urban Development
(11) Tenderness, happiness and locomotion: a healthy door building project in Lioujia	Urban Development
(12) Building a sky garden to enjoy green view	Environmental Protection
Equity	
(1) Tuberculosis-free homeland	Public Health
(2) Upgrading health care services for people living in radiation-contaminated buildings	Public Health
(3) Providing sufficient health care services by using medical buses in Hsin-Chu County	Public Health
(4) No barriers for disabled people	Public Health
(5) Health in Hualien: The influenza prevention project for children and seniors	Public Health
(6) Protection for families with special situation: Orange Daylily micro insurance programs	Social Affairs
(7) My ability is your ability: Programs for the right to read in Nantou	Culture Affairs
(8) Sunshine outside the box, brighten Tainan: Marketing the creative products made by vulnerable people	Labor affairs
(9) Living assistance for new immigrants in Chia-Yi	Civic Affairs
(10) Child wellness protection: The projects providing food and wellness care for children	Education

## Appendix A

**Table A1:** No. of applications and winners and award rates from 2013–2016.

Year	No. of Applications			No. of Winners			Award Rate (%)		
	Total (a)	Health (b)	Non-health (c)	Total (d)	Health (e)	Non-health (f)	Total (g = d/a)	Health ^Ϯ^ (h = e/b)	Non-health ^¶^ (i = f/c)
2013	225	59	166	41	18	23	18.2	30.5	13.9
2014	290	70	220	63	26	37	21.7	37.1	16.8
2015	217	62	155	52	17	35	24.0	27.4	22.6
2016	229	66	163	61	19	42	26.6	28.8	25.8

^Ϯ^ Award rate of health dept. = no. of winners in health department/no. of applications in health department × 100%. ^¶^ Award rate of non-health dept. = no. of winners in non-health departments/no. of applications in non-health departments × 100%.

## References

[1] Tsouros A.D. (1995). The WHO healthy cities project: State of the art and future plans. Health Prom. Internat..

[2] WHO (1997). Twenty Steps for Developing a Healthy Cities Project.

[3] Ståhl T., Wismar M., Ollila E., Lahtinen E., Leppo K. (2006). Health in All Policies: Prospects and Potentials.

[4] WHO (2014). Health in All Policies (HiAP) Framework for Country Action.

[5] Ollila E. (2011). Health in all policies: From rhetoric to action. Scand. J. Public Health.

[6] Ståhl T. (2018). Health in all policies: From rhetoric to implementation and evaluation-the finnish experience. Scand. J. Public Health.

[7] Storm I., Harting J., Stronks K., Schuit A.J. (2014). Measuring stages of health in all policies on a local level: The applicability of a maturity model. Health Policy.

[8] Molnar A., Renahy E., O’Campo P., Muntaner C., Freiler A., Shankardass K. (2016). Using win-win strategies to implement health in all policies: A cross-case analysis. PLoS ONE.

[9] Shankardass K., Renahy E., Muntaner C., O’Campo P. (2015). Strengthening the implementation of health in all policies: A methodology for realist explanatory case studies. Health Policy Plan..

[10] Shankardass K., Muntaner C., Kokkinen L., Shahidi F.V., Freiler A., Oneka G., Bayoumi A.M., O’Campo P. (2018). The implementation of health in all policies initiatives: A systems framework for government action. Health Res. Policy Syst..

[11] Lawless A., Williams C., Hurley C., Wildgoose D., Sawford A., Kickbusch I. (2012). Health in all policies: Evaluating the South Australian approach to intersectoral action for health. Can. J. Public Health.

[12] Freiler A., Muntaner C., Shankardass K., Mah C.L., Molnar A., Renahy E., O’Campo P. (2013). Glossary for the implementation of health in all policies (HiAP). J. Epidemiol. Community Health.

[13] Gase L.N., Pennotti R., Smith K.D. (2013). “Health in all policies”: Taking stock of emerging practices to incorporate health in decision making in the United States. J. Public Health Manag. Pract..

[14] Steenbakkers M., Jansen M., Maarse H., de Vries N. (2012). Challenging health in all policies, an action research study in dutch municipalities. Health Policy.

[15] WHO (2017). Shanghai declaration on promoting health in the 2030 agenda for sustainable development. Health Promot. Int..

[16] Hu S.C., Kuo H.W. (2016). The development and achievement of a healthy cities network in Taiwan: Sharing leadership and partnership building. Glob. Health Promot..

[17] Goumans M., Springett J. (1997). From projects to policy: ‘Healthy cities’ as a mechanism for policy change for health?. Health Promot. Int..

[18] Kingdon J.W., Thurber J.A. (2011). Agendas, Alternatives and Public Policies.

[19] O’Neill M., Simard P. (2006). Choosing indicators to evaluate healthy cities projects: A political task?. Health Promot. Int..

[20] Greer S.L., Lillvis D.F. (2014). Beyond leadership: Political strategies for coordination in health policies. Health Policy.

[21] Sihto M., Ollila E., Koivusalo M. (2006). Principles and challenges of health in all policies. Health in All Policies: Prospects and Potentials.

[22] Barton H., Grant M. (2013). Urban planning for healthy cities. J. Urban Health.

[23] Krech R. (2011). Healthy public policies: Looking ahead. Health Promot. Int..

[24] Koivusalo M. (2010). The state of health in all policies (HiAP) in the european union: Potential and pitfalls. J. Epidemiol. Community Health.

